# EGPDI: identifying protein–DNA binding sites based on multi-view graph embedding fusion

**DOI:** 10.1093/bib/bbae330

**Published:** 2024-07-08

**Authors:** Mengxin Zheng, Guicong Sun, Xueping Li, Yongxian Fan

**Affiliations:** School of Computer Science and Information Security, Guilin University of Electronic Technology, Guilin 541004, China; School of Computer Science and Information Security, Guilin University of Electronic Technology, Guilin 541004, China; School of Computer Science and Information Security, Guilin University of Electronic Technology, Guilin 541004, China; School of Computer Science and Information Security, Guilin University of Electronic Technology, Guilin 541004, China

**Keywords:** protein–DNA binding site prediction, protein language models, multi-view graph embedding fusion, equivariant graph neural network, gated attention mechanism

## Abstract

Mechanisms of protein-DNA interactions are involved in a wide range of biological activities and processes. Accurately identifying binding sites between proteins and DNA is crucial for analyzing genetic material, exploring protein functions, and designing novel drugs. In recent years, several computational methods have been proposed as alternatives to time-consuming and expensive traditional experiments. However, accurately predicting protein-DNA binding sites still remains a challenge. Existing computational methods often rely on handcrafted features and a single-model architecture, leaving room for improvement. We propose a novel computational method, called EGPDI, based on multi-view graph embedding fusion. This approach involves the integration of Equivariant Graph Neural Networks (EGNN) and Graph Convolutional Networks II (GCNII), independently configured to profoundly mine the global and local node embedding representations. An advanced gated multi-head attention mechanism is subsequently employed to capture the attention weights of the dual embedding representations, thereby facilitating the integration of node features. Besides, extra node features from protein language models are introduced to provide more structural information. To our knowledge, this is the first time that multi-view graph embedding fusion has been applied to the task of protein–DNA binding site prediction. The results of five-fold cross-validation and independent testing demonstrate that EGPDI outperforms state-of-the-art methods. Further comparative experiments and case studies also verify the superiority and generalization ability of EGPDI.

## Introduction

The interactions between proteins and deoxyribonucleic acid (DNA) are essential for diverse biological activities and processes [1, 2], including gene expression and regulation, DNA replication, repair, and signal transduction [3, 4]. Accurate identification of protein–nucleic acid interactions is of great significance for understanding protein molecular mechanisms, exploring protein functions [5, 6], and identifying potential drug targets for new drug design [7, 8]. Traditional experimental methods, including X-ray crystallography [9], fast ChIP [10], and electron microscopy [11], are designed to identify the binding modes between proteins and nucleic acids. However, these methods are often time-consuming and expensive. Consequently, there is a strong impetus to develop efficient and accurate computational methods for identifying protein–DNA binding sites.

Existing computational methods for protein–DNA binding site prediction can be broadly classified into two categories, sequence-based methods and structure-based methods, based on the data types utilized. Sequence-based methods primarily learn local patterns of DNA binding from protein sequences before feeding them into various classifiers for prediction. TargetS [12] predicts ligand-binding sites from primary protein sequences using a ligand-specific strategy. SCRIBER [13] uses hidden Markov models to capture long-term dependency characteristics of protein sequences. TargetDNA [14] extracts evolutionary conservation information and predicted solvent accessibility from protein sequences, using a sliding window strategy to learn local patterns of DNA binding. NCBRPred [15] predicts nucleic acid binding residues in proteins using bidirectional Gated Recurrent Units (BiGRUs) [16] to capture global interactions among residues. Although sequence-based methods can be applied to any protein, their lack of crucial protein spatial structure information results in limited prediction accuracy.

In contrast, structure-based methods yield more accurate predictive results by integrating available structural information, and can be categorized into three types: template-based approaches, machine-learning-based approaches, and hybrid approaches. Reliable templates of target proteins are searched using alignment or comparison algorithms, enabling template-based approaches to learn rich genetic information. For instance, COACH-D [17] identifies reliable templates for the query protein from the BioLip dataset. TM-SITE [18] is devised by comparing the structure of consistently distributed subsets of residues, linking them to the binding pockets identified in both the queried and template proteins. COFACTOR [19] identifies template proteins with similar folds and functional sites by threading the target structure through three representative template libraries. Machine learning-based approaches typically rely on protein sequence and spatial structure information to construct computational models. In GraphBind [20], the secondary structure and atomic spatial position information of proteins are encoded into node and edge features of graphs. GraphSite [21] introduces a single representation of the protein generation model AlphaFold2 [22] to predict protein–DNA binding sites. GLMSite [23] utilizes a geometric vector perceptron-based graph neural network (GVP-GNN) to address the protein–DNA binding site prediction task. EquiPNAS [24] employs an equivariant graph neural network as protein encoder. Hybrid approaches meld template-based and machine learning-based approaches. For instance, DNABind [25] combines machine learning methods with template methods, enhancing the accuracy of predicting binding sites. NucBind [26] combines predictions from the template-based method COACH-D and the machine learning-based method SVMnuc [26]. NABind [27], combines deep learning and template modules using sequence and structural descriptors, accurately predicting DNA- and RNA-binding residues. However, template-based methods are heavily dependent on the quality of templates, making them susceptible to inaccuracies. Hybrid-based methods are encumbered by high computational complexity. Furthermore, current computational methods use single-feature representation and single encoder, limiting their ability to extract comprehensive information from complex proteins. Therefore, the accurate identification of protein–DNA binding sites remains an ongoing challenge.

Considering the significant impact of both sequences and local patterns of tertiary structures on protein functional sites [28]. Designing handcrafted features requires sufficient biological knowledge and may lose critical information. Deep learning techniques have demonstrated potential in learning intricate binding patterns from proteins, presenting a solution to the limitations in manual features design [29]. Recently, pretrained protein language models [30–33] have been widely used to generate embeddings for various downstream tasks, such as protein structure prediction [31, 33] and function prediction [30, 32]. Additionally, recent advancements in EGNN [34] have demonstrated its ability to handle spatial translation and rotation invariance of molecules. As an illustration, FABind [35] incorporates an E(3) equivariant graph neural network into the encoder, enhancing the performance of protein and ligand docking prediction. EQGAT [36] confirmed that EGNN outperforms traditional graph neural networks in representing protein structures. Furthermore, the gated attention mechanism [37], dynamically adjusts attention weights and has enhanced the performance of the multi-head attention mechanism [38].

In this work, we propose a novel computational method, EGPDI, aimed at identifying protein–DNA binding sites based on multi-view graph embedding fusion. By converting the binding site prediction task into a graph node classification problem, we map each protein sequence into a graph representation, with amino acids serving as nodes. Node features are enriched by amalgamating handcrafted features with diverse embeddings derived from protein language models. The graph’s topology is articulated through a distance matrix, established by calculating the Euclidean distance between amino acids, subsequently translated into an adjacency matrix. The deeper information extraction process employs both the GCNII module and the EGNN module to extract local and global embeddings, respectively. To tackle the vanishing gradient issue in multi-layer graph neural networks, we innovatively apply initial residual connections and identity mapping in the EGNN module. Finally, an advanced gated multi-head attention mechanism is applied to integrate these embeddings efficiently, capturing important information while addressing the challenge of strong heterogeneity. To our knowledge, this is the first time that multi-view graph embedding fusion has been applied to the task of protein–DNA binding site prediction. Besides, we comprehensively evaluate EGPDI on benchmark datasets and independent test set, and the results show that EGPDI outperforms existing methods. The datasets and the source code of EGPDI are freely available at https://github.com/HaaZheng/EGPDI.

## Materials and methods

### Benchmark datasets

To compare with existing methods, we utilize three widely recognized public datasets. They are the training set (DNA_573_Train) and test set (DNA_129_Test) from GraphBind, which contain 573 proteins and 129 proteins, respectively. And the independent test set (DNA_181_Test) from GraphSite, which contains 181 proteins. The average protein length in DNA_181_Test is about 415 amino acids, compared to 290 in DNA_129_Test. DNA_129_Test has no proteins over 1000 amino acids while DNA_181_Test contains 18 such proteins. A DNA-binding site is defined when the smallest atomic distance between the DNA molecule and the target residue is less than 0.5 Å. Datasets were obtained by selecting proteins with potentially similar biological functions from multiple DNA-protein complexes. Additionally, CD-HIT [39] was used to ensure that no redundant protein with >30% sequence identity within the training set and between the training and test sets. The details of these public datasets are shown in Table 1.

**Table 1:** Summary of the benchmark datasets

Dataset	Proteins	Binding residues	Nonbinding residues	PN_ratio_
DNA_573_Train	573	14 479	145 404	0.100
DNA_129_Test	129	2240	35 275	0.064
DNA_181_Test	181	3208	72 050	0.045

### Problem formalization

In this work, the DNA binding site prediction problem is treated as a graph node classification problem. Each protein sequence is represented as a graph, which is defined as $\mathbf{G}=\left(\mathbf{X},\mathbf{E},\mathbf{A}\right)$. $\mathbf{X}={\left\{{x}_i\right\}}_{i=1,\dots, N}$ and ${x}_i\in{R}^{L\times 6524}$ denote the node feature matrix and the node feature vector of node $i$, respectively, where $L$ represents the length of protein sequence. $A$ is defined as an adjacency matrix with the shape of $N\times N$. And edge feature matrix is defined as $\mathbf{E}=\left\{{e}_{ij}|{\mathbf{A}}_{ij}=1\right\}$, where ${e}_{ij}\in{R}^2$ stands for the edge feature vector between node $i$ and node $j$. ${\mathbf{A}}_{ij}=1$ if the centroid of residue side-chain between node $i$ and node $j$ is less than 17 Å, otherwise, ${\mathbf{A}}_{ij}=0$. This particular distance threshold is derived from the results of our independent cross-validation experiments.

### Node representations

Summarize all node feature representation strategies and obtain a final $6524$-dimensional feature matrix. The details of these node features are shown in Table 2.

**Table 2:** Summary of node features

Features [shape]	Description
PSSM [L,20]	Normalized position-specific scoring matrix (PSSM)
HMM [L,30]	Normalized Hidden Markov Models Matrix (HMM)
One-hot encoding [L,20]	One-hot encodings of 20 amino acid residue types
Atomic features [L,7]	Atomic features of residues (AF)
SS [L,14]	Secondary structure profiles (SS)
MSA [L,256]	Multiple sequence alignment (MSA)
ESM-2 [L,5153]	pLM embeddings from ESM-2 with 15B and 3B parameters
ProtTrans [L,1024]	pLM embeddings from ProtTrans

### Edge representations

For edge features on the graph, two different types of position encodings are computed. The details of these node features are shown in Table 3, where ${d}_e$ denotes the number of edges. Specifically, for each amino acid in the target protein, Euclidean coordinates in three-dimensional space are captured as coordinates features, dedicated to the EGNN module.

**Table 3:** Summary of edge features

Features [shape]	Description
Euclidean distance [${d}_e$,1]	The Euclidean distance between two nodes in three-dimensional space.
Cosine value of angle [${d}_e$,1]	The cosine values of the angle between two residues

### The architecture of EGPDI

**Figure 1:**
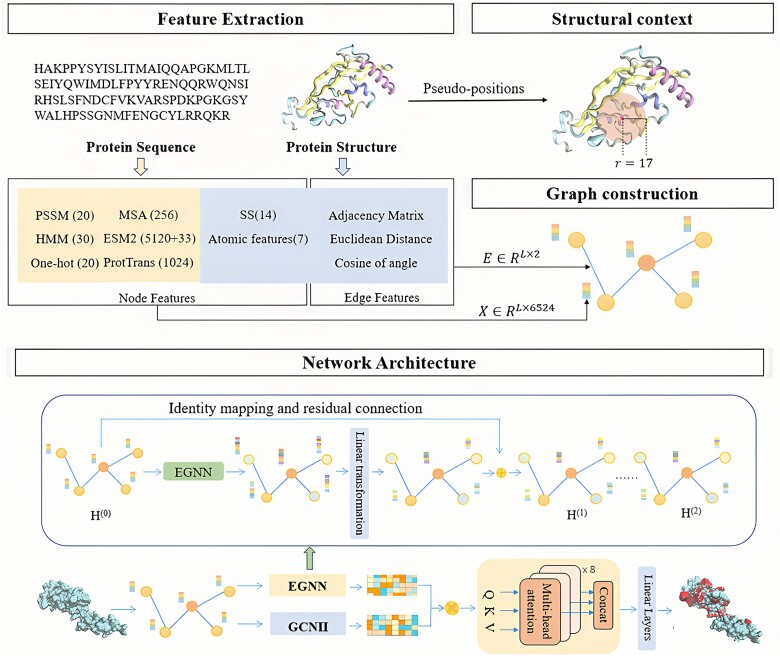
The overall architecture of EGPDI. (1) Feature extraction. PSSM, HMM, one-hot encoding, MSA, ESM-2 embeddings, and ProtTrans embeddings are extracted from protein sequences, while SS and atomic features are extracted from protein structure, collectively forming the node features. Edge features are composed of two types of position encodings, and an adjacency matrix is also generated. (2) Structural context extraction. The structural context of a target residue is determined by a sliding sphere of a predefined radius (*r* = 17 Å) centered at the residue. (3) Graph construction. The node features, edge features, and structural context of a target protein are aligned to construct graph data. (4) Network architecture. The graph data is fed into the GCNII module and the EGNN module, each incorporating initial residual connection and identity mapping. An improved gated multi-head attention mechanism is utilized to fuse two types of deep graph embeddings effectively. Finally, the fused embeddings are passed through the MLP module to obtain the prediction results.

In this work, we propose EGPDI, a protein–DNA binding site prediction method based on a multi-view graph fusion framework that aggregates multi-source information. The overall architecture of EGPDI is shown in Fig. 1. Initially, handcrafted features are combined with the protein language models (pLMs) features as node features, distance matrix and two types of edge features are calculated to construct the topology of the graph. Subsequently, the graph data are separately passed to the GCNII and EGNN modules, which introduce identity mapping and initial residual connection ideas.

Therefore, we obtain two different deeper graph embeddings. An improved gated multi-head attention mechanism is adopted to effectively combine two embeddings, and these embeddings are transmitted to the MLP module to obtain the protein–DNA binding site classification result.

### Graph convolutional networks II

Graph Convolutional Networks (GCN) [40] and their variants, notably GCNII, have shown significant success in graph node classification tasks in recent years. GCNII extends GCN by introducing initial residual connections and identity mapping to effectively tackle the over-smoothing issue [41]. It maintains the core message-passing mechanism, using adjacency and node feature matrices for efficient information propagation and prioritizes neighboring nodes to capture local information effectively in graph embeddings.

In this work, our GCNII module comprises four layers with a hidden dimension of 128.

### Equivariant graph neural network

Equivariant Graph Neural Network (EGNN) is a variant of GNN [42] that introduces coordinate features, distinguishing it from traditional GNNs. By implementing coordinate equivariant transformations, EGNN can capture translation-, rotation-, and reflection-equivariant characteristics within three-dimensional molecules. Therefore, utilizing EGNN to extract protein features can acquire more structural properties. Another distinction from traditional GNNs is EGNN’s capability to process both equivariant and invariant features simultaneously. Multiple equivariant graph convolution layers (EGCL) are stacked to form EGNN. EGCL updates the coordinate features ${x}_i^{l+1}$ and node features ${h}_i^{l+1}$ of the next layer based on the coordinate features ${x}_i^l$, node features ${h}_i^l$, and edge features ${e}_{ij}$ input from the previous layer. The update rule of node coordinate features in EGCL is defined as follows:


(1) \begin{equation*} {m}_{ij}={\varPhi}_e\left({h}_i^l,{h}_j^l,{\left\Vert{x}_i^l-{x}_j^l\right\Vert}^2,{e}_{ij}\right) \end{equation*}

(2) \begin{equation*} C=\frac{1}{M-1},{m}_{ij}\in M \end{equation*}

(3) \begin{equation*} {x}_i^{l+1}={x}_i^l+C\ \sum \limits_{j\ne i}\left({x}_i^l-{x}_j^l\right){\varPhi}_x\left({m}_{ij}\right) \end{equation*}

Initially, the relative distance between node $i$ and node $j$, edge features ${e}_{ij}$ and their node features ${h}_i^l,{h}_j^l$ are aggregated through the MLP operation of edges ${\varPhi}_e$. $C$ denotes a constant factor chosen as $1/M-1$, where $M$ is the number of graph nodes.

The aggregated information ${m}_{ij}$ is processed by ${\varPhi}_x$, an MLP operation of nodes. The node features of node $i$ from the previous layer and the sum of its relative coordinate differences with all the other nodes are taken into account for updating the node in the next layer.

In addition to incorporating the entire graph nodes when updating coordinate features, EGNN also integrates the entire graph information in node features and edge features. Unlike GCNII, which tends to capture local information, EGNN tends to capture global information. The aggregated information ${m}_i$ of node $i$ is collected from all the other nodes. ${\varPhi}_h$ denotes the MLP operation of node. The updating process of node features is as follows:


(4) \begin{equation*} {m}_i=\sum \limits_{j\ne i}{m}_{ij} \end{equation*}

(5) \begin{equation*} {h}_i^{l+1}={\varPhi}_h\left({h}_i^l,{m}_i\right) \end{equation*}

In this work, our EGNN module comprises two layers with a hidden dimension of 512.

### EGNN with initial residual and identity mapping

Increasing the number of layers in the model may lead to overfitting in GCN. However, reducing the depth of the model can result in insufficient features extracted from neighbors. Inspired by GCNII, we introduced the concept of initial residual connection and identity mapping to EGNN. The update rule of EGCL is defined as follows:


(6) \begin{equation*} {\mathrm{H}}^{\left(l+1\right)}=\sigma \left(\left(\left(1-\alpha \right)P{H}^{(l)}+\alpha{H}^{(0)}\right)\left(\left(1-{\beta}_l\right){I}_n+{\beta}_l{W}^{(l)}\right)\right) \end{equation*}

(7) \begin{equation*} {\beta}_l=\log \left(\frac{\lambda }{l}+1\right) \end{equation*}

where $\alpha, \beta$ are hyperparameters, $P$ is the normalized adjacency matrix. ${H}^{(l)},{H}^{(0)}$ denote the node feature matrix of the $l\mathrm{th}$ layer and the initial node feature matrix, respectively. Based on EGNN, two improvements are implemented: (i) Initial residual connection: adding the initial node feature matrix ${H}^{(0)}$ and smoothing matrix $P{H}^{(l)}$. (ii) Identity mapping: adding the weight matrix of the $l\mathrm{th}$ layer ${W}^{(l)}$ with an identity map ${I}_n$. In this way, even if stack multiple layers of EGCL, at least part of the original node features can be retained in the final feature embedding, effectively mitigating overfitting.

### Improved gated multi-head attention module

To focus on more important features, we introduce the attention mechanism [38] to fuse the two graph embeddings from the GCNII module and EGNN module. Initially, the graph embeddings from different perspectives are merged. The combined graph embeddings $f\in{R}^{L\times 640}$ are treated as the query matrix $Q$, key matrix $K$, and value matrix $V$. However, the self-attention mechanism may overly focus on itself; hence, to distribute attention across different feature spaces, a multi-head attention mechanism is employed. By calculating the attention weight for each head, the attention distribution in each feature space is determined.

The attention weight is calculated as shown:


(8) \begin{equation*} {\mathrm{Attention}}_i=\mathrm{softmax}\left(\frac{\left(Q{W}_i^Q\right)\left(K{W}_i^k\right)}{\sqrt{d_k}}\right) \end{equation*}

(9) \begin{equation*} {\mathrm{head}}_i={\mathrm{Attention}}_i\left(V{W}_i^v\right) \end{equation*}

${W}_i^Q,{W}_i^k,{W}_i^v$ represent the learnable matrices for the query, key, and value matrices, respectively. And ${\mathrm{Attention}}_i$ denotes the attention matrix with a size of $L\times L$, where $i=1,\dots, H$. In this work, $H=16$. In order to further dynamically adjust the output of global information, a gated mechanism [37] similar to LSTM [43] is introduced on the multi-head attention mechanism. The implementation of the gated mechanism is shown as follows:


(10) \begin{equation*} G=\sigma \left(f{W}^G+{b}^G\right) \end{equation*}

(11) \begin{equation*} {h}_i^{\mathrm{gated}}=G\ \mathrm{e}\ {\mathrm{head}}_i \end{equation*}

(12) \begin{equation*} {h}_{\mathrm{gated}}=\mathrm{concat}\ \left({h}_i^{\mathrm{gated}},\mathrm{L},\kern0.75em {h}_H^{\mathrm{gated}}\right)\mathrm{W} \end{equation*}

Through the gated mechanism, the output information ${h}_{gated}$ is obtained, where ${W}^G,{b}^G,W$ are all learnable parameters, and $\odot$ represents the vector element product. However, due to the high complexity of protein structure, a single-gated multi-head attention mechanism may not capture adequate information. Therefore, the outputs of multiple independent gated multi-head attention mechanisms are concatenated to obtain a more comprehensive representation. The final output of the gated multi-head attention mechanism module is calculated as shown, where $N=8$ and $H\in{R}^{L\times 640\times 8}$.


(13) \begin{equation*} H=\mathrm{concat}\ \left({h}_{\mathrm{gated}}^{\mathrm{i}}\right),\kern0.5em i=1,K,N \end{equation*}

## Results and discussion

The proposed method undergoes objective evaluation using five-fold cross-validation (5-CV), and repeats 10 times to ensure reliable predictive results. To assess and compare model performance objectively, widely used performance evaluation metrics including Specificity, Precision, Recall, F1-score (F1) and Matthews correlation coefficient (MCC) are employed, the formulas are calculated in the supplementary material.

### Feature ablation experiments

To determine the optimal feature combination, feature combination methods are divided into three categories: handcrafted features, pLMs features and merged features. The experimental results are shown in Table 4.

**Table 4:** Performance of different features on training set using five-fold cross-validation

Dataset	Features	Spe	Rec	Pre	F1	MCC	AUC	AUPR
DNA_573_Train	Handcrafted features	0.936	0.460	0.445	0.447	0.391	0.856	0.423
	pLMs features	**0.969**	0.571	0.651	0.608	0.573	0.938	0.626
	Merged features	0.964	**0.677**	**0.668**	**0.666**	**0.637**	**0.956**	**0.690**

*Note*: AlphaFold2 predicted protein structures used for evaluation. Bold fonts are the best results.

Handcrafted features consist of PSSM, HMM, One-hot encoding, SS and AF, while pLMs features include ESM-2 embeddings, ProtTrans embeddings, and MSA. Merged features represent a fusion of handcrafted features and pLMs features. Table 4 illustrates that, with the exception of Specificity, which exhibits no improvement, other metrics for merged features show enhancements. Specially, compared to handcrafted features and pLMs features, the model utilizing the merged features demonstrates an increase in MCC by 24% and 6.4%, AUC by 10% and 1.8%, and AUPR by 26.7% and 6.4%, respectively. This notable improvement can be attributed to the diverse functional and structural properties captured by pLMs features derived from large protein datasets and the rich genetic information contained in handcrafted features.

### Effectiveness of basic modules

**Figure 2:**
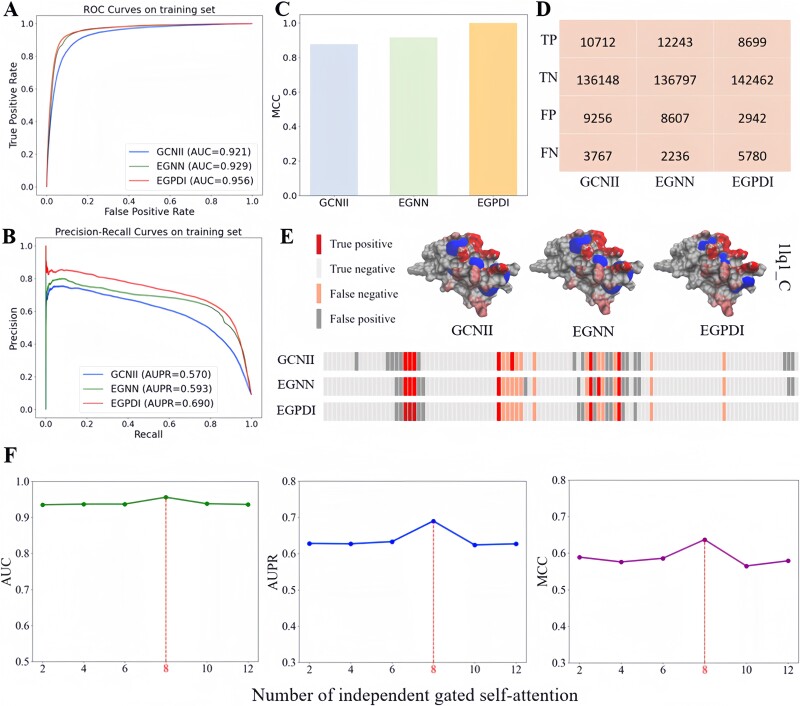
The effectiveness analysis of the basic modules in EGPDI. The ROC curve (A) and PR curve (B) illustrate the performance of the EGNN module, GCNII module and EGPDI on the training set. Additionally, the MCC histogram (C) and confusion matrix (D) provide further insights. An illustrative example of protein–DNA site prediction is depicted in (E). Moreover, the impact of the number of independent gated multi-head attention mechanisms on model performance is analyzed in (F).

In this section, we conducted a comprehensive evaluation of the basic modules in EGPDI. The results of the experiment are shown in Fig. 2 and Table 5 below.

**Table 5:** Performance of different modules on training set using five-fold cross-validation

Dataset	Module	Spe	Rec	Pre	F1	MCC	AUC	AUPR
DNA_573_Train	GCNII	0.942	0.667	0.540	0.599	0.559	0.921	0.570
	EGNN	0.935	**0.741**	0.533	0.615	0.584	0.929	0.593
	EGPDI	**0.964**	0.677	**0.668**	**0.666**	**0.637**	**0.956**	**0.690**

*Note*: Values in bold represent the best performance.

Table 5 presents the comprehensive performance of the GCNII module, EGNN module and EGPDI across all evaluation metrics. EGPDI demonstrates superior performance across multiple evaluation metrics compared to EGNN and GCNII. Interestingly, GCNII module alone yields inferior results compared to utilizing the EGNN module alone. This difference can be attributed to the inherent characteristics of each module: GCNII primarily captures local information through neighbor node sampling, while EGNN comprehensively captures global information by sampling the entire graph. The integrated approach of EGPDI leverages the strengths of both modules, resulting in enhanced predictive performance. As depicted in Fig. 2A and B, EGPDI achieves better performance on both the ROC curve and PR curve. The confusion matrix in Fig. 2D reveals that for the DNA_573_Train dataset, EGPDI accurately predicts 151 161 sites, surpassing EGNN by 2121 and GCNII by 4301. Furthermore, the visual analysis in Fig. 2E demonstrates that EGPDI reduces the blue coverage in the three- dimensional map, while reducing the gray area in the residue position diagram, further supporting the effectiveness of EGPDI in reducing false positives and enhancing prediction accuracy. Moreover, the examination of the number of independent gated multi-head attention mechanisms in Fig. 2F shows that employing multiple independent gated multi-head attention mechanisms can effectively enhance model performance. The optimal performance was achieved when the number reaches 8.

### Effectiveness of different embedding fusion

**Figure 3:**
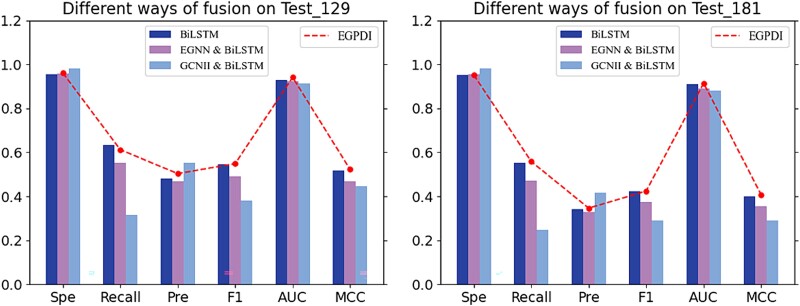
Performance of EGPDI and three variants: BiLSTM, EGNN & BiLSTM, GCNII & BiLSTM on test sets DNA_129_Test and DNA_181_Test.

In this section, we validated the superiority of the EGPDI model architecture based on multi-view graph embedding fusion on the test sets. The superior performance of the EGPDI model architecture is confirmed, as shown in Table 6. To further validate the fusion of multi-view graph embedding between the EGNN module and the GCNII module, we designed three methods and conducted a comparative analysis with EGPDI on both test sets. The comparative results are depicted in Fig. 3.

**Table 6:** Performance of EGPDI on two test sets

Dataset	Specificity	Recall	Precision	F1	AUC	MCC
DNA_129_Test	0.961	0.612	0.503	0.549	0.941	0.522
DNA_181_Test	0.952	0.558	0.346	0.424	0.914	0.407

BiLSTM [44], a widely utilized model in predicting protein–DNA binding sites, is employed as the baseline for our comparative analysis with GCNII and EGNN. To systematically compare the performance of EGPDI, we developed three variants. BiLSTM model comprises two bidirectional LSTM layers, only utilizing node features to calculate the probability of binding sites. The second variant, EGNN & BiLSTM, passes the node features to a two-layer BiLSTM and feeds the graph data to an EGNN module with four EGCL layers Similarly, the third variant, GCNII & BiLSTM, processes the node features through BiLSTM and feeds the graph data to GCNII individually to acquire different embeddings. Notably, all other components of the model architecture remain consistent across all variants.

As shown in Fig. 3, on DNA_129_Test, the GCNII & BiLSTM variant exhibits a decrease in performance across multiple metrics, including Rec, F1, AUC, and MCC. Additionally, the EGNN & BiLSTM variant consistently demonstrates inferior performance across all evaluation metrics compared to EGPDI. The experimental results of DNA_181_Test exhibit similar trends, as detailed in Supplementary Table S1. Overall, the experimental results indicate that the fusion of BiLSTM-based and graph-based embeddings does not lead to performance improvement. The proposed method outperforms all variants across key metrics. By obtaining two graph-based embeddings from multiple perspectives, EGPDI effectively mitigates potential heterogeneity and redundancy in information among different types of embeddings.

### Performance comparison with other methods

We compare EGPDI with five existing methods on test set DNA_129_Test and independent test set DNA_181_Test. Table 7 records the detailed experimental results.

**Table 7:** Performance comparison with state-of-the-art methods on two test sets

Dataset	Method	Specificity	Recall	Precision	F1	AUC	MCC
DNA_129_Test	COACH-D	0.955	0.328	0.318	0.323	0.712	0.279
	NucBind	**0.964**	0.322	0.366	0.343	0.809	0.304
	GraphSite	0.950	0.566	0.423	0.441	0.912	0.425
	GLMSite	0.816	**0.848**	0.287	0.405	0.918	0.412
	EquiPNAS	0.956	0.516	0.471	0.462	0.919	0.443
	EGPDI	0.961	0.612	**0.503**	**0.549**	**0.941**	**0.522**
DNA_181_Test	COACH-D	**0.971**	0.239	0.266	0.251	0.668	0.220
	NucBind	0.959	0.288	0.240	0.262	0.798	0.227
	GraphSite	0.958	0.454	0.343	0.345	0.892	0.332
	GLMSite	0.805	**0.829**	0.209	0.311	0.899	0.334
	EquiPNAS	0.958	0.436	0.346	0.366	0.907	0.353
	EGPDI	0.952	0.558	**0.346**	**0.424**	**0.914**	**0.407**

*Note*: The results of other methods come from the paper NABind [27] proposed by Zhen *et al*. Values in bold represent the best performance.

EGPDI demonstrates significant improvements in key metrics, such as F1, AUC, and MCC, on both DNA_129_Test and DNA_181_Test compared to the suboptimal method. Specifically, on DNA_129_Test, EGPDI shows enhancements of 8.7%, 2.2%, and 7.9% in F1, AUC, and MCC, respectively, while on DNA_181_Test, improvements of 5.8%, 0.7%, and 5.4% are observed. The performance of EGPDI on the MCC metric on the two test sets is shown in Supplementary Fig. S2. However, the performance of EGPDI on the Spe and Rec metrics appears to be average, possibly due to their high sensitivity to threshold selection. It is worth noting that the COACH-D method and the NucBind method suffer from poor prediction performance when reliable templates are unavailable, and the GraphSite method which uses graph transformer architecture cannot handle proteins with complex structures well and loses important structural prior knowledge. Additionally, the GLMSite method only uses ProtTrans embeddings as node features, which leads to incomplete protein characterization. EGPDI’s success in overcoming these limitations and achieving superior performance lies in its template-independent approach and comprehensive characterization of proteins through diverse representation features. Additionally, the incorporation of the EGNN module enables the retention of crucial structural prior knowledge, while the enhanced gated multi-head attention mechanism efficiently fuses graph embeddings, further improving the performance of model.

### Case studies

**Figure 4:**
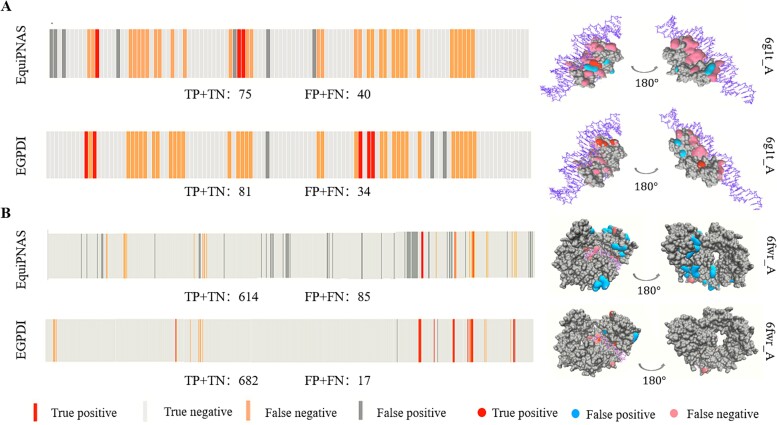
The three-dimensional map and residue position diagram of protein 6g1t_A (A) and 6fwr_A (B).

In this section, we conduct case studies to verify EGPDI’s capability to recognize unknown protein–DNA binding sites. Protein 6g1t_A and 6fwr_A are chosen from DNA_129_Test and DNA_181_Test, respectively. These two representative examples of protein–DNA site prediction made by EGPDI and EquiPNAS are plotted in Fig. 4. Protein 6g1t_A consists of 115 residues, while protein 6fwr_A consists of 699 residues. Figure 4A shows that EGPDI accurately predicts six more residues on protein 6g1t_A compared to EquiPNAS. Figure 4B demonstrates that EGPDI accurately predicts 68 more residues on protein 6fwr_A compared to EquiPNAS. These findings indicate the efficacy of EGPDI in enhancing the prediction performance of binding sites. Moreover, a detailed analysis of the sequence diagrams indicates a notable reduction in the number of false positives (FP) when employing the proposed method. The three-dimensional images of proteins6g1t_A and 6fwr_A illustrate the distribution of prediction results for EquiPNAS and EGPDI. Specifically, in the three-dimensional image of EGPDI, the blue and pink regions exhibit smaller areas, whereas the red region appears more prominent.

## Conclusion

Accurately identifying binding sites between proteins and DNA is essential for designing novel drugs and understanding biological processes. Owing to the limitations in protein feature representation and encoder design, current methods still have the potential for prediction improvement. In this study, we propose a novel computational method called EGPDI for predicting protein–DNA binding sites. Firstly, we encode protein molecules into graphs, incorporating both handcrafted features and pLMs embeddings as node features. Additionally, we calculate the Euclidean distance and the cosine values of the angles between adjacent nodes as edge features. Subsequently, we utilized the EGNN module and the GCNII module, both incorporating initial residual connections and identity mapping mechanisms, to independently learn graph embeddings. Then, the acquired graph embeddings are integrated using an enhanced gated multi-head attention mechanism and then forwarded to the MLP module to compute the probabilities of nodes being binding sites. Experimental results on two test sets show the significant superiority of EGPDI over existing methods. Further ablation experiments and case studies also validate the generalization ability of our approach. We summarize that the superiority of EGPDI is mainly attributed to the following reasons: (i) the combination of handcrafted features and pLMs embedding enables a more comprehensive characterization of protein sequences and structural information from different perspectives. (ii) The EGNN module enables to capture global information and preserves the translation-, rotation-, and reflection-equivariant characteristics of protein. In addition, the GCNII module, which learns local information, is integrated with the EGNN module to extract global features from different perspectives. (iii) Utilizing multiple independent gated multi-head attention mechanisms for graph embedding fusion allows EGPDI to concentrate on more important features and diminish information redundancy.

Despite the promising results achieved by our method, it still has some shortcomings. Firstly, our approach is influenced by the predictive quality of AlphaFold2. However, this impact has been somewhat alleviated by adding manually designed sequence-based features and pLMs embeddings. Secondly, only using scalar edge features may not fully capture the complex geometric properties of protein molecules. To address this issue, future work will explore the incorporation of vector-based edge features. Thirdly, we will consider collecting DNA information, because numerous studies have also shown that DNA structural information plays a crucial role in predicting these binding sites [45–47]. Lastly, we expect to extend our feature representation and multi-view graph embedding fusion strategy to other binding site prediction problems.

**Key Points**

- EGPDI is a protein–DNA interaction site predictor based on multi-view graph embedding fusion, which treats protein–DNA interaction site prediction as a classification task of graph nodes.
- The combination of handcrafted features and pLMs embedding enables a more comprehensive characterization of proteins.
- The basic EGNN module with initial residual and identity mapping captures high-order translation-, rotation-equivariant characteristics within proteins by introducing coordinate equivariant transformations.
- GCNII primarily captures local information through neighbor node sampling, while EGNN comprehensively captures global information by sampling the entire graph.
- An advanced gated multi-head attention mechanism is applied to integrate these embeddings efficiently, capturing extensive information while addressing the issue of information redundancy.

## Supplementary Material

EGPDI_Supplementary_Materials_bbae330_V1

## Data Availability

The datasets and the precomputed features used in this study are available at https://github.com/HaaZheng/EGPDI.

## Author Biographies

**Mengxin Zheng** received her Bachelor of Engineering degree from Beijing Language and Culture University, China, in 2022. She is currently pursuing her Master's degree in Computer Science and Technology at Guilin University of Electronic Technology, China. Her research interests include deep learning, bioinformatics, and pattern recognition.

**Guicong Sun** received his BS degree from Taiyuan University of Science and Technology, China, in 2020. He is currently pursuing his Ph.D. degree in computer science with the Guilin University of Electronic Technology, China. His research interests include deep learning, pattern recognition, and bioinformatics.

**Xueping Li** received her Bachelor of Computer Engineering degree from Guangxi Normal University for Nationalities, China, in 2022. She is currently pursuing her Master's degree in Computer Science and Technology at Guilin University of Electronic Technology, China. Her research interests include deep learning, medical image segmentation, and interpretable machine learning.

**Yongxian Fan** received the Ph.D. degree from the Institute of Image Processing and Pattern Recognition, Shanghai Jiao Tong University. He is currently a professor with the School of Computer Science and Information Security, Guilin University of Electronic Technology, China. His research interests include artificial intelligence, data analysis, machine learning, pattern recognition, and bioinformatics.
