# Supplementary material for: EGPDI: identifying protein–DNA binding sites based on multi-view graph embedding fusion
Source: Brief Bioinform. 2024 Jul 8;25(4):bbae330. doi: 10.1093/bib/bbae330 (PMC11229037; doi:10.1093/bib/bbae330)
Supplement: EGPDI_Supplementary_Materials_bbae330_V1 [file egpdi_supplementary_materials_bbae330_v1.docx]

**EGPDI: identifying Protein-DNA binding sites based on multi-view graph embedding fusion**

## Supplementary Materials

**Supplementary Table S1.** Comparison of EGPDI with designed methods on two test sets

| **Dataset** | **Method** | **Spe** | **Rec** | **Pre** | **F1** | **AUC** | **MCC** |
| --- | --- | --- | --- | --- | --- | --- | --- |
| DNA_129_Test | BiLSTM | 0.956 | **0.633** | 0.480 | 0.545 | 0.930 | 0.518 |
|  | GCNII& BiLSTM | **0.981** | 0.316 | **0.533** | 0.380 | 0.914 | 0.445 |
|  | EGNN& BiLSTM | 0.957 | 0.554 | 0.468 | 0.492 | 0.924 | 0.468 |
|  | EGPDI | 0.916 | **0.612** | 0.503 | **0.549** | **0.941** | **0.522** |
| DNA_129_Test | BiLSTM | 0.953 | 0.554 | 0.343 | 0.422 | 0.911 | 0.401 |
|  | GCNII& BiLSTM | **0.982** | 0.249 | 0.417 | 0.291 | 0.879 | 0.288 |
|  | EGNN& BiLSTM | 0.954 | 0.472 | 0.330 | 0.373 | 0.891 | 0.355 |
|  | EGPDI | 0.952 | **0.558** | **0.346** | **0.424** | **0.914** | **0.407** |


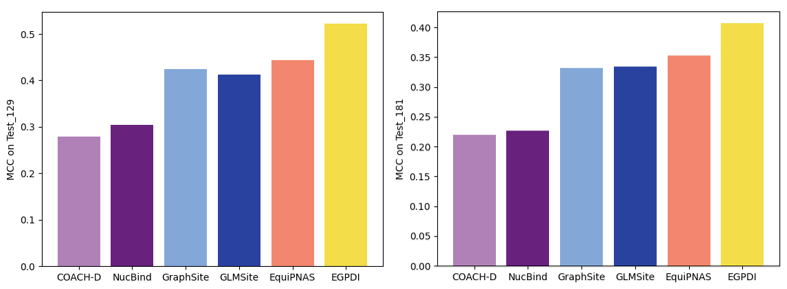


**Supplementary Figure S2.** Comparative results of EGPDI and the latest methods on two test sets.

**AlphaFold2**

To generate protein spatial structures, AlphaFold2 is commonly utilized, offering accurate predictions with atomic-level precision, even in the absence of homologous templates. It leverages various input databases, including sequence databases such as UniRef90, BFD, Mgnify cluster, and structure databases like PDB and PDB70. During training, AlphaFold2 transfers protein bio-physicochemical knowledge obtained through multiple sequence alignment technology into the Evoformer-based deep learning algorithm. The average error of predicted protein structures typically falls within the range of 1Å. In this study, AlphaFold2 is employed to generate predicted structures for all proteins within the training and test sets to facilitate subsequent analysis.

**Experiment details**

The model training settings are established based on existing research experience, and model parameters are obtained through grid search. Specifically, two layers of EGCL are stacked to acquire a 512-dimensional graph embedding, while four layers of GCNII yield a 128-dimensional graph embedding. Both the EGNN module and the GCNII module share the hyperparameters of the initial residual connection and identity mapping. These two types of graph embeddings are effectively fused by using eight independent 16-head gated multi-attention mechanisms. Additionally, the learning rate is equal to 0.0001, cross-entropy loss function is utilized, and Adam is selected as the optimizer. Furthermore, dropout layers with a value of 0.3 are applied multiple times in the model to prevent overfitting.

**Performance evaluation**

$$\text{Specificity =}\frac{\text{TN}}{\text{TN+FP}}$$

$$\text{Precision}=\frac{\text{TP}}{\text{TP}+\text{FP}}$$

$$\text{F1}=2\cdot\frac{\text{Precision}\times\text{Recall}}{\text{Precision+Recall}}$$

$$\text{MCC}=\frac{\text{TP}\times\text{TN}-\text{FN}\times\text{FP}}{\sqrt{\left(\text{TP}+\text{FP}\right)\times\left(\text{TP}+\text{FN}\right)\times\left(\text{TN}+\text{FP}\right)\times\left(\text{TN}+\text{FN}\right)}}$$

where true positives (TP) and true negatives (TN) denote the number of binding and non-binding sites identified correctly, and false positives (FP) and false negatives (FN) denote the number of incorrectly predicted binding and non-binding sites, respectively. In addition, the area under the receiver operating characteristic curve (AUC) and the area under the precision-recall curve (AUPR) are also utilized to evaluate model performance.

**Node features**

*Evolutionary conversation profiles*

The evolutionary conversation profiles include position-specific scoring matrix (PSSM) and Hidden Markov Models matrix (HMM). PSSM utilizes the alignment tool PSI-BLAST, searches the NCBI’s non-redundant database for homologous sequences with three iterations and $E-value<10^{-3}$, which applies heuristic algorithms and dynamic programming [1] . The size of PSSM is $L\times20$ and every element $x$ in the PSSM is normalized between 0 and 1 by Equation.

$$\bar{x}=\frac{1}{1+e^{-x}}$$

HMM employs HHblits to search against the uniclust30 database [2] for the query sequences [3]. The size of HMM is $L\times30$ and every element $a$ in the HMM is also normalized between 0 and 1 by Equation.

$$\bar{a}=\frac{a}{10000}\text{ }$$

*One-hot encoding*

One-hot encoding represents the 20 amino acids constituting a protein with unique binary codes. Each protein can be converted into a one-hot encoded matrix with the size of $L\times20$.

*Atomic features of residues*

Atomic features of residues (AF), which refers to the work of GraphBind. For target residue, seven kinds of features of each atom belonging to the residue are extracted. The size of AF matrix is $L\times14$. They are atom mass, B-factor, whether it is a residue side-chain atom, electronic charge, the number of hydrogen atoms bonded to it, whether it is in a ring, and the van der Waals radius of the atom. $\left\{f_{s,t}\right\}_{s=1,\ldots,7,t=1,\ldots,N_{a}}$denotes the original atomic features of a residue, where $f_{s,t}$ stands for the $s\text{th}$ feature of $t\text{th}$ atom and $N_{a}$ stands for the number of atoms belonging to the residue. Since different residues may have different numbers of atoms, the atomic features are further processed. Finally, we get seven kinds of atomic features for each residue$\left\{x_{s}\right\}_{s=1,\ldots,7}$. The processing process is as follows:

$$x_{s}=\frac{1}{N_{a}}\left(\sum_{t=1}^{t=N_{a}} f_{s,t}\right)$$

*Secondary structure profiles*

The secondary structure profiles (SS) use DSSP [4,5] to generate the SS matrix with the size of $L\times14$. In details, this matrix includes eight states of secondary structure: B (residue in isolated β-bridge), E (extended strand, participates in β-ladder), G ($3^{10}$-helix), H (α-helix), I (π-helix), S (bend), T (H-bonded turn), five columns of bond and torsion angles and one column of residue water-exposed surface.

*Multiple sequence alignment*

The extraction of Multiple sequence alignment (MSA) refers to the work of EquiPNAS. By running the tool ColabFold [6] and the complementary method of ColabFold, the input sequence is encoded into a dictionary. The MSA is the first line of output information with the size of $L\times256$.

*Protein language model representations*

ProtTrans is a transformer-based method, pre-trained on UnirefR50 dataset to learn rich evolutionary knowledge from protein sequences. The size of ProtTrans embeddings is $L\times1024$. The latest protein language model ESM-2 trains model on large-scale protein sequence databases, learning the evolutionary rules and the sequence-structure-function relationship of proteins. In detail, esm2_t48_15B_UR50D pre-trained model was employed to get the embedding of $L\times5120$, and esm2_t36_3B_UR50D was employed to get the embedding of $L\times33$.

**Edge features**

*Euclidean distance*

The euclidean distance$d_{ij}$between two nodes in three-dimensional space can be calculate by Equation, where $x_{i},x_{j}$ denotes the coordinates of node $i$ and node $j$ , respectively.

$$d_{ij}=\left\|x_{i}-x_{j}\right\|$$

*Cosine value of the angle between residues*

The cosine values of the angle between two residues $\cos\left(\theta_{ij}\right)$ can be calculate by Equation, where $p_{i},p_{j}$ and $p_{0}$ denotes the coordinates of node $i$ , node $j$ and sphere center, respectively. The vector between node $i$ and sphere center can be represented as $\overrightarrow{p_{0}p_{i}}$, similarly, $\overrightarrow{p_{0}p_{j}}$ represents the vector between node $j$ and sphere center.

# REFERENCES

1. Altschul S. Gapped BLAST and PSI-BLAST: a new generation of protein database search programs. Nucleic Acids Res. 1997; 25:3389–3402

2. Mirdita M, von den Driesch L, Galiez C, et al. Uniclust databases of clustered and deeply annotated protein sequences and alignments. Nucleic Acids Res. 2017; 45:D170–D176

3. Remmert M, Biegert A, Hauser A, et al. HHblits: lightning-fast iterative protein sequence searching by HMM-HMM alignment. Nat. Methods 2012; 9:173–175

4. Kabsch W, Sander C. Dictionary of protein secondary structure: Pattern recognition of hydrogen‐bonded and geometrical features. Biopolymers 1983; 22:2577–2637

5. Touw WG, Baakman C, Black J, et al. A series of PDB-related databanks for everyday needs. Nucleic Acids Res. 2015; 43:D364–D368

6. Mirdita M, Schütze K, Moriwaki Y, et al. ColabFold: making protein folding accessible to all. Nat. Methods 2022; 19:679–682

7. Huang Z, Xu W, Yu K. Bidirectional LSTM-CRF Models for Sequence Tagging. 2015;
